# The Application of Nanomaterials in the Treatment of Pancreatic-Related Diseases

**DOI:** 10.3390/ijms252313158

**Published:** 2024-12-07

**Authors:** Jing Ma, Xue Li, Chunru Wang

**Affiliations:** 1 Key Laboratory of Molecular Nanostructure and Nanotechnology, Institute of Chemistry, Chinese Academy of Sciences, Beijing 100190, China; majing23@iccas.ac.cn (J.M.); crwang@iccas.ac.cn (C.W.); 2 University of Chinese Academy of Sciences, Beijing 100049, China

**Keywords:** nanomaterials, pancreas, diabetes, pancreatic cancer, pancreatitis

## Abstract

Pancreatic diseases, typically including pancreatic cancer, pancreatitis, and diabetes, pose enormous threats to people’s lives and health. To date, therapeutics with high therapeutic efficacy and low side effects are still challenging. With the development of nanotechnology, nanomaterials have successfully been applied in pancretic disease treatment. Here, we first introduce the diversity of nanomaterials and the effects of their different physicochemical properties on pancreatic function. Following this, we analyze the potential of nanomaterials to enhance pancreatic targeting by overcoming the challenges of traditional delivery methods through surface modifications, structural adjustments, and optimized drug loading. Then, we introduce the application of structurally optimized nanomaterials to pancreatic-related diseases. For instance, on pancreatic cancer (as drug delivery platforms, for the promotion of radiation therapy, and as multifunctional tools), pancreatitis (as drug delivery systems, anti-inflammatory and anti-fibrotic agents), and diabetes (as insulin delivery carriers, for protecting pancreatic β cells, and for improving insulin resistance). Through analysis of the progress of current research, we summarize how nanomaterials can enhance treatment efficacy while minimizing side effects. Finally, we look forward to the prospects of nanomaterials in pancreatic disease treatment.

## 1. Introduction

The pancreas is a crucial gland in the human body primarily responsible for secreting digestive enzymes and hormones, such as insulin, which are essential for maintaining normal digestive and metabolic functions [1]. Pancreatic diseases, including pancreatic cancer, pancreatitis, and diabetes, pose significant health risks [2]. These diseases are not only difficult to diagnose early, often being detected at advanced stages, but are also challenging to treat. Pancreatic cancer remains one of the deadliest cancers, with mortality rates nearly matching its incidence due to a combination of biological complexity and late-stage diagnosis [3]. The disease’s pathophysiology, marked by rapid tumor growth, invasiveness, and a unique tumor microenvironment with a dynamic assortment of extracellular matrix components, fosters resistance to conventional treatments such as chemotherapy and radiation. Furthermore, this microenvironment suppresses immune response, reducing immunotherapy efficacy [4]. Given these obstacles, pancreatic cancer poses a global health challenge, highlighting an urgent need for early detection methods and innovative, patient-specific therapies.

Pancreatitis encompasses both acute and chronic forms, each presenting distinct clinical challenges. Chronic pancreatitis is a complex fibroinflammatory condition characterized by recurrent episodes of pancreatic inflammation, leading to significant fibrosis. This process results in chronic pain, progressive exocrine and endocrine insufficiency, reduced quality of life, and decreased life expectancy. The incidence and prevalence of chronic pancreatitis are increasing, yet no curative treatment currently exists [5]. In contrast, acute pancreatitis is an unpredictable, often life-threatening disease. Its prognosis largely hinges on the presence of organ failure and the risk of secondary infection in pancreatic or peripancreatic necrosis. Severe cases of acute pancreatitis continue to show high mortality rates [6].

Diabetes is a metabolic disease characterized by hyperglycemia that derives from reduced insulin production by pancreatic islet cells or the body’s insensitivity to insulin. The progression of diabetes is intricately linked to islet cell damage, with these cells playing a pivotal role in insulin production and glucose regulation [7]. Such cellular impairment undermines the body’s ability to maintain proper blood glucose levels, leading to chronic hyperglycemia and, subsequently, the onset of diabetes. Over time, diabetes is frequently accompanied by various complications, including cardiovascular disease, neuropathy, nephropathy, and retinopathy—each posing significant health risks and deeply impacting quality of life. Although advancements have been made in diabetes management, current pharmacological treatments predominantly focus on glycemic control and symptom alleviation rather than providing a definitive cure. Therefore, the effective treatment of pancreatic diseases remains a central focus of current medical research.

Nanotechnology, as an emerging technology, shows great potential in biomedical fields. Nanotechnology refers to the construction and manipulation of matter at the nanoscale [8]. The sizes of conventional nanomaterials are usually between 1 and 100 nanometers, and, according to their size effects, nanomaterials usually exhibit a high surface area-to-volume ratio, quantum effects, surface effects, and surface functionalization properties [9]. These characteristics endow nanomaterials with the potential for widespread application in diagnostic imaging, disease treatment, and tissue engineering. Particularly, nanomaterials have been widely used in the treatment of pancreatic diseases. Several nanomaterials have been used as ideal carriers for targeted drug delivery systems, enabling them to reach the pancreas through the circulatory system and release drugs under specific conditions [10]. For example, polymers responding to γ-glutamyl transpeptidase-loaded camptothecin can actively infiltrate throughout the tumor tissue through transcytosis and treat pancreatic cancer [11]. Lipid-coated mesoporous silica nanoparticle-loaded irinotecan showed high efficacy and reduced toxicity, promoting the first-line application of irinotecan [12]. Additionally, some nanomaterials (fullerene, gold, selenium-based nanoparticles) possess excellent biological effects for treating pancreatic diseases, such as ROS scavenging, anti-inflammatory and immunomodulatory abilities [13,14]. Moreover, the tunability of nanomaterials allows for the modification of surface properties or the combination of bioactive molecules, achieving highly specific biological functionalization, which provides the possibility for personalized medicine [15]. Therefore, nanomaterials could have the potential to improve the therapeutic efficacy and reduce the side effects of pancreatic diseases.

This article aims to explore the applications of nanomaterials in treating pancreatic diseases, particularly focusing on the summary of nanomaterial design and modification according to the characterization of nanoparticles, the pharmacokinetic profiles via different administration routes, and the pathological features of specific diseases. In detail, we first introduce the diversity of nanomaterials and the effects of their different physicochemical properties on pancreatic function and overcoming conventional delivery barriers via surface-modified and structural optimization of nanomaterials. Then, we describe the applications of nanomaterials in pancreatic disease treatment, such as in pancreatic cancer, pancreatitis, and diabetes. Lastly, the emerging trends of nanomaterial application in precision medicine, the development of multifunctional nanomaterials, the integration of gene editing with nanotechnology, the development of immuno-nanomaterials, and the integration of the microbiome and nanotechnology are also prospected. This study on the interactions between nanomaterials and pancreatic diseases is expected to drive the advancement of medical technology and improve the survival rate and quality of life of patients. Ultimately, we hope that this review provides precise guidance and valuable references for researchers in related fields and that it paves the way for the clinical application of nanomaterials for pancreatic diseases in the future.

## 2. Nanomaterial-Enhanced Pancreatic Enrichment

**Figure 1:**
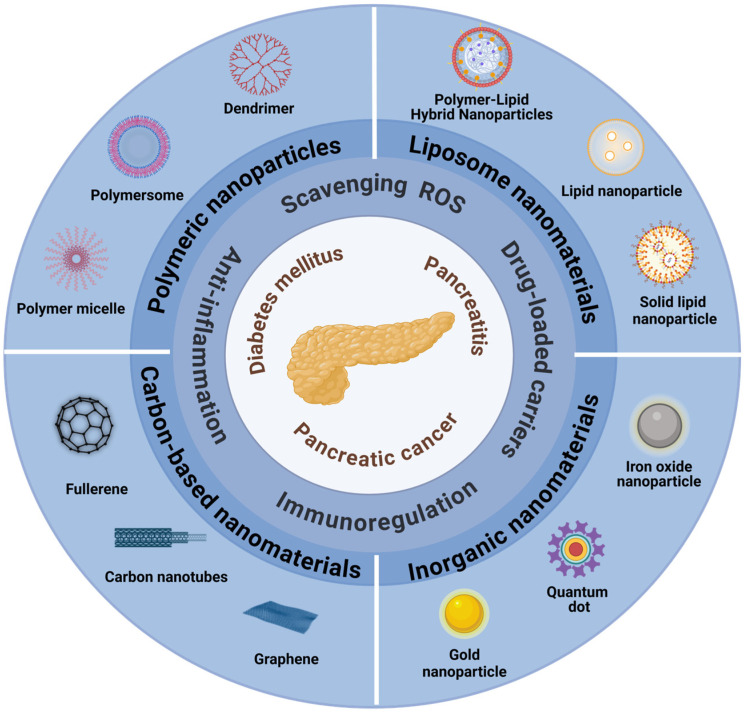
The application of nanomaterials in the treatment of pancreatic-related diseases.

The diverse nature of nanomaterials enables their broad application in disease therapy. Common nanomaterials include inorganic nanomaterials, polymeric nanoparticles, liposome nanomaterials, and carbon-based nanomaterials [16]. Specifically, inorganic nanomaterials, including gold, quantum dots, silica, and iron oxide nanoparticles, are extensively utilized due to their ROS scavenging ability, mesoporous structures, and magnetism. Polymeric nanomaterials include various forms, such as dendrimers, polymer micelles, and polymeric vesicles, such as poly(lactic-co-glycolic acid) (PLGA) and chitosan, which are frequently used in drug delivery systems due to their high biocompatibility and biodegradability [17]. Liposomes, a form of lipid bilayer nanomaterial, mimic cell membranes and possess superior drug encapsulation abilities, making them widely used in drug delivery [18]. Carbon-based nanomaterials enhance drug delivery efficiency and cell penetration owing to their unique mechanical and electrical properties [19]. Particularly, fullerenes and their derivatives, with their stable nanostructure and chemical properties, effectively scavenge reactive oxygen species (ROS), thereby alleviating oxidative damage, inflammatory response, and immune dysfunction [20,21,22]. The above nanomaterials, with the principles of small size, large surface area, and easy functionalization, allow them to treat pancreatic diseases through multiple mechanisms. Understanding the properties of these nanomaterials and their interactions with the pancreas enables the development of more precise and effective solutions for treating pancreatic diseases (as summarized in Figure 1).

### 2.1. The Physicochemical Properties of Nanomaterials Affect Pancreatic Function

Nanomaterial interactions with pancreatic cells mainly involve cellular uptake and intracellular transport. Pancreatic cells mainly internalize nanoparticles through phagocytosis, clathrin-mediated endocytosis, caveolin-mediated endocytosis, clathrin/caveolae-independent endocytosis, and micropinocytosis [23]. Once inside the cell, nanoparticles work with certain biological effects or release their payload, such as drugs or cytokines, to exert therapeutic effects [24]. Nanomaterial transport and distribution in the pancreas depend on their physicochemical properties and interactions with the biological environment [25]. The shape of nanomaterials and their particle size can significantly affect their uptake efficiency by cells or tissues. A study reported that ∼50 nm is the optimum size for nanoparticles to achieve the highest cellular uptake in certain cells [26]. Hence, Zhou et al. prepared PEG–PLGA nanoparticles with different size distributions (NP60, NP150, and NP300), showing that NP150 displayed enhanced pancreas accumulation with higher fluorescence intensity than NP60 and NP300 in acute pancreatitis (AP) models, which was extremely likely due to ELVIS-mediated accumulation [27]. Similarly, Qiang et al. prepared mesoporous silica nanoparticles (MSNs) with different diameters (60, 150, and 300 nm) loaded with the fluorescent dye IR780 and investigated the distribution of those in rats of mild AP (MAP) or severe AP (SAP) [28]. They showed that MSN150 accumulated in the pancreas and ascites of a rat model of AP to a much greater extent than MSN60 or MSN300, and there were lower accumulations of MSNs in the pancreas in the SAP than in the MAP. Another study demonstrated that rod-shaped nanoparticles underwent lower cellular uptake than spherical ones, possibly deriving from the longer membrane wrapping time [29]. Apart from particle size and shape, the surface charge also influences the tissue permeability and biodistribution of nanomaterials. A study compared the zwitterionic embellished lipid (including anionic embellished lipid and cationic embellished lipid), finding that the lipid nanoparticles containing cationic embellished lipids most effectively reduced off-target luciferase expression in the liver and spleen [30,31]. In other words, the nanoparticles with a positive surface charge are more likely to bind to negatively charged cell membranes, thereby increasing their uptake efficiency. The design of nanomaterials should not only take into account the size, shape, and surface charge but also maximize the avoidance of bulk removal by metabolic organs. The metabolic rate and clearance mechanisms vary among different materials. The absorption efficiency and therapeutic effect of nanomaterials can only be enhanced by the enrichment of a sufficient amount of therapeutic agent in the diseased area. Nanomaterials’ metabolic pathways primarily involve the liver, kidneys, and reticuloendothelial system (RES) [32]. Metal nanoparticles are often metabolized by the liver, while some polymeric nanomaterials are excreted through the kidneys [33]. As for pancreatic diseases, nanomaterials are enriched in the pancreas through blood circulation and then finally gradually metabolized and cleared in the pancreas [34].

Interactions between nanomaterials and pancreatic cells can lead to a range of biological effects, such as cytotoxicity or antioxidative stress and anti-inflammatory responses. A nanomaterial’s cytotoxicity is closely related to its chemical composition, surface modification, and exposure duration. On the one hand, metal oxide nanoparticles (such as ferroferric oxide or arsenic trioxide) may induce oxidative stress in pancreatic cells, leading to cellular damage or apoptosis [35]. On the other hand, certain nanomaterials, such as carbon nanotubes, graphene derivatives, and fullerenes, are considered to have lower cytotoxicity, making them a suitable carrier or therapeutic agent in treating pancreatic diseases [36,37,38]. Additionally, the unique properties of nanomaterials enable them to significantly influence the pancreatic microenvironment, particularly through interactions with the extracellular matrix, vascular system, and immune cells, thereby affecting the physiological functions of the pancreas. Most typically, the nanomaterials can increase the accumulation of antitumor drugs in pancreatic tumor sites by altering the permeability and retention effect (EPR effect) of tumor tissues. This phenomenon helps to increase the local concentration of drugs and reduce systemic side effects [39]. Nanomaterials can also carry various bioactive molecules, such as anti-inflammatory drugs or immune modulators, effectively altering the immune microenvironment of the pancreas [40].

### 2.2. Enhancing Pancreatic Targeting via Different Administration Routes

Nanomaterials are administered into the body via different routes and distributed to the organs, interacting with cells and tissues with certain biological functions. Various administration routes—such as oral administration, intravenous injection, and intraperitoneal injection—can significantly affect the interactions between nanomaterials and the pancreas, thereby influencing treatment efficacy and safety. Modifying or structurally optimizing nanomaterials to address the challenges associated with various drug delivery methods allows nanomaterials to achieve enhanced bioavailability and targeting (as summarized in Table 1), thereby providing more effective therapeutic options for patients with pancreatic diseases.

#### 2.2.1. Oral Administration

Orally administered nanomaterials present an attractive approach for the treatment of pancreatic diseases. The development of oral nanomedicines focuses on overcoming the physiological barriers posed by the gastrointestinal (GI) tract to ensure the efficient delivery of active compounds to the pancreas [41]. Recently, oral nanomaterials targeting the pancreas have been developed and applied in the treatment of pancreatic diseases.

**Figure 2:**
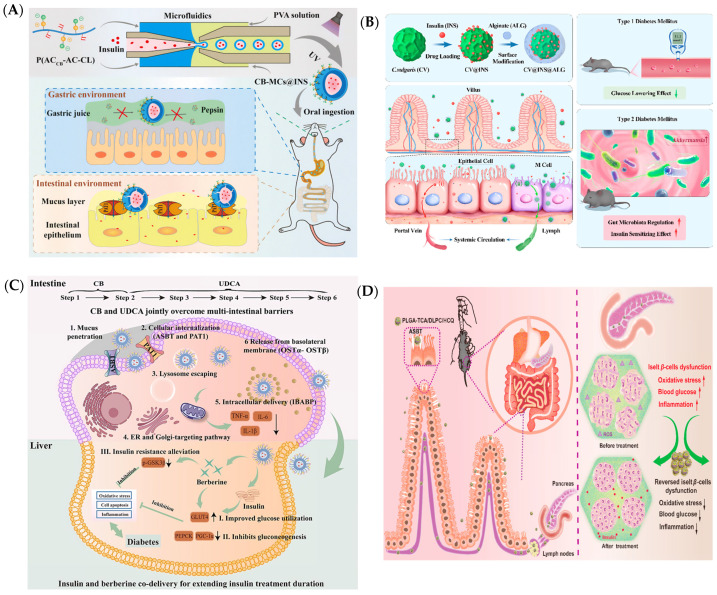
Oral nano drug delivery to the pancreas. (**A**) CB–MCs@INS via the combination of microfluidics and UV-crosslinking to improve oral insulin delivery [43]. (**B**) pH-responsive CV@INS@ALG for oral insulin delivery [44]. (**C**) ACU@BI by liver-targeted zwitterionic nanoparticles to overcome multi-intestinal barriers and extend insulin treatment duration [45]. (**D**) PLGA-TCA/DLPC/HCQ combined with apic-sodium-dependent bile acid transporters mediated intestinal uptake and lymphatic transport of drugs [46].

Orally administered nanoparticles encapsulating clinical drugs or biologics are designed to protect the active substances from degradation in the harsh, acidic environment of the stomach and facilitate their absorption in the intestines [42]. Research indicates that cross-linked amphoteric ion microcapsules (CB-MCs@INS) made from carboxybetaine (CB)-modified poly(acrylate-co-caprolactone) copolymers can enhance the intestinal absorption and pancreatic targeting of nanoparticles (Figure 2A). The introduction of CB can increase the affinity of nanoparticles for intestinal epithelial cells, facilitating their passage through the intestinal barrier and thereby enhancing their bioavailability. The combination of microfluidics and UV cross-linking can improve the delivery of oral insulin. The CB-MC@INS microcapsules have a high drug loading capacity (>40%) and can protect insulin from acid degradation in harsh gastric environments [43]. An insulin delivery system based on Chlorella vulgaris (CV) was cross-linked with sodium alginate (ALG), resulting in a microalgae-based oral insulin delivery strategy (CV@INS@ALG) (Figure 2B). The CV@INS@ALG nanomaterials could overcome gastrointestinal barriers, protecting insulin from harsh gastric conditions and enabling a pH-responsive drug release in the intestine [44]. The oral delivery of berberine through zwitterionic nanoparticles has shown potential in extending treatment duration by overcoming intestinal barriers and protecting the drug from degradation. Ursodeoxycholic acid (UDCA) and carboxy betaine (CB) modification endowed ACU@BI with the ability to penetrate mucus and target the apical and basolateral side of enterocytes, respectively, which jointly overcame multi-intestinal barriers (Figure 2C). In vivo experiments indicated that the sustained release and prolonged circulation of insulin and berberine in the intestinal tract synergistically maintained the hypoglycemic effect (approximately 10 h) and avoided the risk of hypoglycemia after oral administration of ACU@BI with lowered dosage (insulin: 25 U/kg). Moreover, the oral co-delivery of berberine by ACU@BI reversed insulin resistance mainly via not only the facilitation of the AMPK/AKT/IRS-1 signaling pathway but also the suppression of GSK3-β activity that was found for the first time, thereby ultimately extending the duration of insulin therapy [45]. PLGA-TCA/DLPC/HCQ, taurocholic acid (TCA)-modified poly(lactic-co-glycolic acid) (PLGA) was employed to achieve the pancreas location, hydroxychloroquine (HCQ) was loaded to execute therapeutic efficacy, and 1,2-dilauroyl-sn-glycero-3-phosphocholine (DLPC) was introduced as a stabilizer together with a synergist (Figure 2D). In vitro and in vivo results have proven that PLGA-TCA/DLPC/HCQ reversed the pancreatic islets damage and dysfunction, thus impeding hyperglycemia progression and restoring systemic glucose homeostasis via only one administration every day. In terms of mechanism, PLGA-TCA/DLPC/HCQ ameliorated oxidative stress, remodeled the inflammatory pancreas microenvironment, and activated the PI3K/AKT signaling pathway without obvious toxicity [46].

Despite the promising prospects of oral administration in pancreatic disease treatment, the main challenge lies in improving the bioavailability and pancreatic targeting of nanomaterials. Future directions may include the development of more gastrointestinal stabilized nanocarriers and optimizing their distribution within the pancreas.

#### 2.2.2. Intravenous Administration

Intravenous injection is one of the primary routes for delivering nanomaterials to the pancreas. Through intravenous injection, nanomaterials can directly enter the bloodstream and quickly reach the pancreatic site. As intravenous injection can bypass the degradative effects of the gastrointestinal tract, it is an effective method for achieving the efficient delivery of nanomaterials. By loading therapeutic elements, such as antioxidants, anti-inflammatory drugs, or anticancer molecules, onto nanomaterials, these elements can be specifically delivered to pancreatic parts, protecting them from free radical and inflammatory damage and thereby relieving the progression of pancreatic diseases [47].

However, nanomaterials are easily captured by the RES, resulting in many of them being removed and little enrichment at the pancreatic site. Therefore, many targeted delivery techniques are being developed to enhance the accumulation of nanomaterials in the pancreas. Targeted pancreas delivery techniques mainly include passive targeting and active targeting. Passive targeting leverages the enhanced permeability and retention (EPR) effect of pancreatic tumors, deriving from the loose endothelial junctions, aberrant and defective vascular architecture, imperfect lymphatic drainage, and enhanced expression levels of proteins and factors related to vascular permeability [48], allowing nanomaterials to accumulate at the tumor site [49], increasing the local concentration of antitumor drugs [50]. Nanoparticles such as polyethylene glycol (PEG)-modified liposomes and biomimetic membrane nanoparticles have been reported to enhance EPR effects and increase the accumulation of loadings in pancreatic tumor cells [51]. Additionally, some physical methods, like ultrasound, light, magnetism, and so on [52,53,54], have also been found to promote the EPR effects.

**Figure 3:**
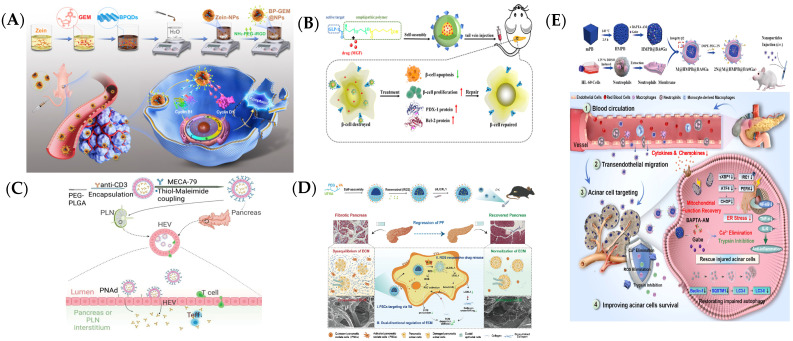
Intravenous injection for delivering nanomaterials to the pancreas. (**A**) BP-GEM@NPs combining bioactive black phosphorus [56]. (BP) and gemcitabine (GEM) for pancreatic cancer treatment. (**B**) MGF/GDPP polymeric nanoparticles actively targeting the pancreas for diabetes [57]. (**C**) The working mechanism of MECA79-anti-CD3-NP for treatment of T1DM [58]. (**D**) The expected mechanism of LR-SSVA toward ECM normalization for the regression of PF [60]. (**E**) Therapeutic mechanism in a mouse model of AP induced by sodium taurocholate retrograde infusion [60].

Active targeting involves modifying specific molecules (e.g., antibodies, peptides, aptamers, or small-molecule ligands) on the surface of nanoparticles to enhance their binding affinity to pancreatic cell receptors, thereby improving drug delivery specificity [55]. To increase tumor targeting and reduce side effects, Geng et al. designed a tumor-specific nanoplatform (BP-GEM@NPs) that co-encapsulates BPQDs and GEM into Zein-NPs, which further conjugates a tumor-penetrating peptide, iRGD, on their surface (Figure 3A). The iRGD-modified zein nanoparticles co-loaded with BP quantum dots (BPQDs) and GEM were designed and prepared as a targeted nanoplatform (BP-GEM@NPs). After intravenous injection, the in vivo distribution and pharmacokinetics results demonstrated that BP-GEM@NPs showed excellent tumor-targeting capability and significantly prolonged the blood circulation time [56]. Glucagon-like peptide 1 (GLP-1), an active targeting agent to the pancreas, was immobilized on the block copolymer polyethyleneglycol–polycaprolactone (PEG-PCL), delivering mangiferin (MGF) to pancreatic β cells for the enhanced protection of pancreatic β cells and type 1 diabetes mellitus (T1DM) efficacy (Figure 3B) [57]. MECA79 mAb, which can recognize high endothelial venules (HEVs), was modified on the surface of poly(lactic-co-glycolic acid)–poly(ethylene glycol) nanoparticles (NPs), realizing the efficient delivery of anti-CD3 mAb to the PLNs and pancreata of NOD mice and the significant reversal of T1DM (Figure 3C) [58]. Reactive oxygen species (ROS)-responsive nanoparticles have been developed to target and remodel the extracellular matrix (ECM), a key factor in pancreatic diseases. The polymer micelles (LR-SSVA) were functionalized with vitamin A (VA) for targeted delivery to pancreatic stellate cells (PSCs encapsulated resveratrol (RES), and adsorbed the RNA interference drug (siLOXL1) on their surface (Figure 3D) [59]. These micelles are engineered to release their cargo specifically in response to a high ROS environment, promoting the degradation of fibrotic ECM and normalizing tissue structure. This targeted delivery not only enhances therapeutic efficacy but also reduces systemic side effects by concentrating the therapeutic action within a disordered pancreas. A novel therapeutic system was developed using acid-responsive hollow mesoporous Prussian blue nanoparticles (HMPBs) wrapped with neutrophil membranes and surface-modified with the N,N-dimethyl-1,3-propanediamine moiety (Figure 3E). This system co-delivers a calcium chelator (BAPTA-AM) and a trypsin inhibitor (gabexate mesylate), showing precise targeting of pancreatic acinar cells in AP mouse models. The treatment effectively reduces inflammation, restores cell health, and significantly improves survival rates, offering a promising upstream strategy for managing AP with clinical translation potential [60].

However, targeted delivery techniques also have challenges, including ensuring the specificity of the targeting molecules and avoiding immune system recognition. Future directions may include the development of multifunctional nanomaterials that possess both targeting and immune evasion capabilities, thereby improving the therapeutic efficacy.

#### 2.2.3. Intraperitoneal Injection

Intraperitoneal injection refers to the method of directly injecting nanomaterials into or near the specifically targeted organ in therapeutic applications. This route of administration offers notable advantages in terms of efficiency and precision, particularly in the treatment of pancreatic diseases, such as pancreatic cancer, diabetes, and pancreatitis. Through delivering therapeutic agents directly to the affected site, local injection maximizes the drug concentration in the lesion area, thereby enhancing therapeutic outcomes while minimizing the systemic side effects commonly associated with other forms of drug delivery [61].

**Figure 4:**
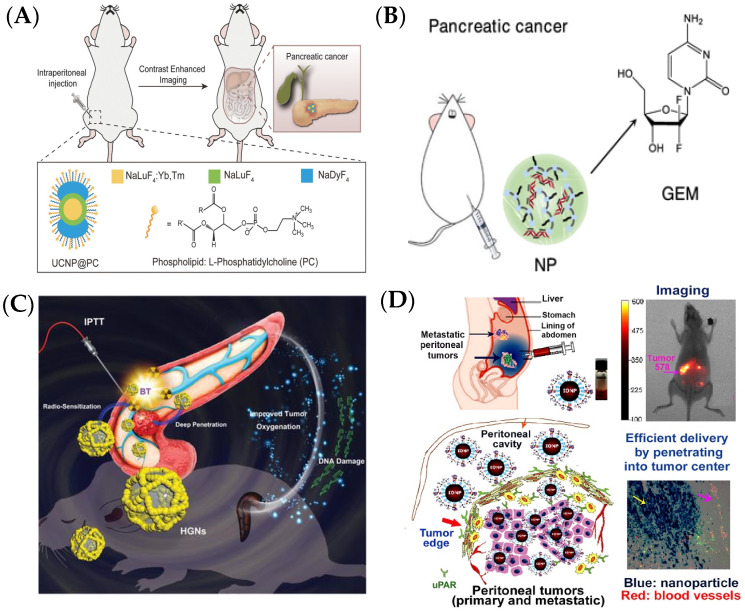
Intraperitoneal injection for delivering nanomaterials to the pancreas. (**A**) UCNP@PC enhances pancreatic cancer accumulation through intraperitoneal injection [65]. (**B**) PAMD-CHOL/siPLK1 nanoparticles enhance anticancer activity of GEM in pancreatic cancer [66]. (**C**) Biodegradable HGNs-mediated IPT–BT synergistic therapy for pancreatic cancer [67]. (**D**) Intraperitoneal injection delivery of uPAR-targeted theranostic IONPs [68]. The yellow arrow indicates the blue nanoparticles, and the pink arrow indicated red blood vessels.

An intraperitoneal injection is applied to extensively deliver nanoparticles to the pancreas [62]. A study reported that, after intraperitoneal injection, most fullerene nanoparticles were distributed in the pancreas; however, there was little accumulation in the pancreas both from intravenous and intragastric administration [63]. Using intraperitoneal injection, gadofullerene nanoparticles could efficiently suppress orthotopic pancreatic cancer and significantly extend the survival rate of tumor-bearing mice [64]. Yuan et al. designed a phosphatidylcholine-modified NaLuF4:Yb,Tm/NaLuF4/NaDyF4 upconversion nanoparticle (UCNP@PC) and found that there was a 16-fold improvement in the imaging efficacy by using intraperitoneally administered UCNP@PC compared to the intravenous approach (Figure 4A) [65]. Nanoparticles formed by cholesterol-modified PAMD (a polymeric CXCR4 antagonist) and siPLK1 (polo-like kinase 1) can strongly accumulate in primary and metastatic tumors, showing strong synergism with gemcitabine for therapeutics of pancreatic cancer (Figure 4B) [66]. A biodegradable honeycomb gold nanoparticle (HGN) was utilized as an internal photothermal agent and radiosensitizer. The HGN-mediated interventional photothermal–brachytherapy (IPT-BT) synergistic therapy achieved a high tumor inhibition rate of 96.6% in SW1990 orthotopic pancreatic tumor mice after intraperitoneal injection (Figure 4C) [67]. Additionally, nanoparticles modified with targeted molecules can efficiently enhance the accumulation of nanoparticles in the pancreas through intraperitoneal administration. Gao et al. reported that urokinase plasminogen activator receptor (uPAR)-targeted magnetic iron oxide nanoparticles (IONPs) could extensively accumulate in peritoneal tumors for imaging-guided therapeutics with up to 17% of the total injected nanoparticles, three-fold higher than with intravenous delivery (Figure 4D) [68].

Studies have shown that ionizable lipids can be used to construct lipid nanoparticles (LNPs) that carry a positive charge at physiological pH, enhancing their fusion and endocytosis with target cell membranes. Melamed et al. reported a strategy for delivering mRNA potently and specifically to the pancreas using lipid nanoparticles. They found that intraperitoneal administration improved pancreatic mRNA delivery compared to intravenous injection. They also elucidated the mechanisms underlying the dependence of pancreatic mRNA delivery on horizontal gene transfer using peritoneal macrophage exosome secretion, enabling gene therapies for intractable pancreatic diseases, such as diabetes and cancer [30].

In addition, osmotic pump implantation in the peritoneal cavity has been used for continuous pancreas delivery [69]. A study made 7- and 14-day micro-osmotic pumps with glucagon solutions at the concentrations necessary for delivery and implanted them in the peritoneum of mice, normalizing the blood glucose levels and correcting the islet morphology of the transgenic intercross PC2−/− [70]. Ling et al. implanted osmotic pumps loaded with GABA into the peritoneal space, finding that exogenous GABA administration did not significantly alter islet cell mass in non-diabetic CD-1 mice in the short term [71].

The primary advantage of intraperitoneal injection is its ability to deliver therapeutic agents with high precision and efficiency. By administering nanomaterials directly to the pancreas, nanomaterials can achieve a higher concentration of the drug at the target site compared to systemic administration. This approach reduces the likelihood of adverse effects, as the rest of the body is exposed to lower drug levels. Additionally, nanomaterials can be engineered to offer the controlled or sustained release of drugs, ensuring prolonged therapeutic action at the lesion site [72]. However, this method also carries risks, such as complex procedures and injection site infections. Therefore, the clinical application of intraperitoneal injection requires the integration of precise imaging technologies to ensure both accuracy and safety. The application of nanomaterials in the treatment of pancreatic diseases depends not only on their unique physicochemical properties but also on the mode of administration. Different administration routes can significantly affect the distribution, metabolism, and interaction of nanomaterials within the pancreas. With the continuous development of nanotechnology, the combined application of different administration routes will further promote the personalized and precise treatment of pancreatic diseases.

## 3. The Application of Nanomaterials in the Treatment of Pancreatic Diseases

### 3.1. The Application of Nanomaterials in Pancreatic Cancer Treatment

Pancreatic cancer is a malignant tumor that is challenging to diagnose in its early stages, associated with a poor prognosis, and characterized by a significantly high mortality rate. In recent years, nanomaterials have demonstrated considerable potential for the treatment of pancreatic cancer, attributed to their superior physicochemical properties (as summarized in Table 2). These nanomaterials not only facilitate targeted drug delivery, thereby increasing the concentration of drugs at the tumor site but also enhance the efficacy of radiotherapy and chemotherapy. Furthermore, certain nanomaterials exhibit antitumor activity and immune-modulating capabilities, offering novel strategies and renewed hope for the treatment of pancreatic cancer.

#### 3.1.1. Nanomaterials as Drug Delivery Systems

Improving drug delivery systems is crucial for enhancing therapeutic efficacy in pancreatic cancer treatment. Traditional chemotherapy drugs, such as gemcitabine and paclitaxel, though somewhat effective, are limited by low bioavailability and systemic toxicity, restricting their therapeutic outcomes [73]. Nanomaterials (such as lipid-based, polymeric, and inorganic nanoparticles) can encapsulate these drugs within nanoparticles, facilitating targeted delivery to pancreatic cancer cells and thus reducing drug accumulation in normal tissues and minimizing side effects [74]. Poly lactic-co-glycolic acid nanoparticles (PLGA NPs) conjugated to a tumor-specific MUC1 antibody, TAB004, were used as nanocarriers for targeted delivery into human pancreatic ductal adenocarcinoma (PDA) cell lines in vitro and in PDA tumors in vivo. The research results showed that TAB004-conjugated paclitaxel (PTX) nanocarriers were significantly more cytotoxic in vitro against PDA cells than their non-conjugated counterparts [75]. Abraxane, an albumin-bound paclitaxel nanoparticle formulation, is superior to conventional paclitaxel preparations. Research indicates that Abraxane-derived HSA was taken up into endothelial cells or tumor cells by a mechanism different from normal endogenous albumin. This novel cellular transport pathway, mediated by gp family proteins, differs from the pathway utilized by innate albumin. These findings elucidate the mechanisms underlying the targeted tumor delivery and antitumor efficacy of Abraxane, providing a scientific basis for developing a new albumin-based drug delivery strategy through receptors specific to denatured albumin. Recently, Abraxane was approved by China’s National Medical Products Administration (NMPA) for use in combination with gemcitabine as a first-line treatment for metastatic pancreatic cancer [76]. Moreover, Liu et al. developed a novel supramolecular dendrimeric nano-system to carry the anticancer drug doxorubicin (FAD-Dox), demonstrating potent anticancer activity and effectively overcoming the heterogeneity of drug response and resistance observed in primary cultured tumor cells derived from pancreatic ductal adenocarcinoma (PDAC) patients. This dendrimer nanodrug is constructed from a fluorinated amphiphilic dendrimer, which self-assembles into micellar nanostructures that encapsulate doxorubicin with high loading efficiency. The unique properties of this dendrimeric nano-system, including its fine nanosize, stable formulation, and acid-promoted drug release mechanism, allow for effective accumulation in tumor tissues. At the same time, this realizes deep penetration within the tumor microenvironment and a rapid drug uptake and release profile in cells. As a result, the nano-system not only exhibits significant anticancer activity but also achieves complete suppression of tumor growth in patient-derived xenograft models [77]. In addition to relying on aptamers, nanomaterials with positively charged and modified membrane-penetrating peptides are also a means to achieve precise release of therapeutic components. The peptide p5RHH, which encapsulates and delivers siRNA, possesses endosomolytic properties, allowing it to effectively penetrate cells and release siRNA into the cytoplasm. By designing KRAS-siRNA nanoparticles as a positively charged system, they attracted negatively charged tumor cells, and this charge difference was utilized to enhance the specific targeting capability of the nanoparticles [78]. Nanomaterial-loaded immune checkpoint inhibitor therapies can enhance the immune response and reduce side effects, such as mesoporous nanomaterials Cu_2_MoS_4_ (CMS)/PEG loaded with PD-L1 inhibitor BMS-1 and CXCR4 inhibitor plerixafor to form the nanodrug CMS/PEG-B-P. Subcutaneous tumorigenicity experiments in C57BL/6 mice verified that CMS/PEG-B-P had an inhibitory effect on the growth of tumors and remodeling of the tumor immune microenvironment, including the infiltration of CD4+ and CD8+ T cells and the polarization of macrophages, as well as the reduction of immunosuppressive cells. Meanwhile, CMS/PEG-B-P was found to have effects on the release of cytokines in the tumor immune microenvironment [79].

#### 3.1.2. Nanomaterials Enhanced Radiation Therapy

Nanomaterials have emerged as a powerful tool in enhancing the efficacy of radiation therapy for pancreatic cancer treatment. Due to the aggressive nature of pancreatic cancer, conventional radiation therapy often struggles to deliver sufficient doses to the tumor site without damaging the surrounding healthy tissues. Nanomaterials, particularly metal-based nanoparticles such as gold nanoparticles, have shown great potential as radiosensitizers. These nanoparticles enhance the absorption of radiation in cancer cells, thereby increasing the effectiveness of the treatment. Studies like those by Kesharwan et al. have demonstrated how gold nanoparticles, when delivered to the pancreatic tumor site, significantly improve the precision and efficacy of radiotherapy [80]. Nanoparticles were designed to deeply penetrate into tumor tissues, enhancing radiation absorption and leading to more effective tumor cell destruction while minimizing the radiation dose to healthy tissues. Cerium oxide nanoparticles (CONPs) are currently being tested in pre-clinical trials as an adjuvant to sensitize pancreatic cancer cells to RT and protect normal tissues from the harmful side effects [81]. Additionally, intracellular transcytosis can enhance the penetration of nanomedicines to deep avascular tumor tissues, but strategies that can improve transcytosis are limited. The study to design a collagenase conjugating transcytosis nanoparticle (Col-TNP) can dissociate into collagenase and cationized gold nanoparticles in response to tumor acidity, which enables their ECM tampering ability and active transcytosis in tumors. The breakage of ECM further enhances the active transcytosis of cationized nanoparticles into deep tumor tissues as well as the radiosensitization efficacy of pancreatic adenocarcinoma [82]. Additionally, Sanja et al. explored the protective effects of fullerenol C_60_(OH)_24_, which showed promise in reducing radiation-induced damage in healthy tissues [83]. This promotes combination therapy by using nanomaterials to not only enhance tumor-targeting radiation but also mitigate adverse effects. In summary, nanomaterials serve dual functions in radiation therapy; they enhance the radiation dose absorbed by the tumor cells, and certain nanomaterials may also protect the surrounding healthy tissues from radiation damage. This dual action helps to improve the therapeutic index of radiotherapy in pancreatic cancer, making treatment both safer and more effective.

#### 3.1.3. Multifunctional Nanomaterials Promote Theranostics

The development of multifunctional nanomaterials presents new avenues for treating pancreatic cancer, enabling both drug delivery and a combination of diagnostic and therapeutic roles. Incorporating chemotherapy drugs, radiation therapy enhancers, and imaging probes into one nanocarrier, these “theranostic” nanomaterials offer greater precision and efficiency in pancreatic cancer treatment. This combination of diagnostics and therapy significantly improves the precision of treatment and has the potential to greatly enhance therapeutic outcomes for pancreatic cancer patients. Combining intraperitoneal administration with phosphatidylcholine-camouflaged NaLuF4:Yb,Tm/NaLuF4/NaDyF4 upconversion nanoparticles (UCNP@PC) enables enhanced dual-modal imaging (upconversion luminescence/magnetic resonance imaging) for orthotopic pancreatic cancer. Modified with phosphatidylcholine—a major component of cell membranes—the optimized nanostructures demonstrate excellent biocompatibility and are rapidly excreted via the bile pathway following intraperitoneal administration. GFNPs penetrate vascular endothelial cells and smooth muscle cells, achieve labeling of up to 30% of tumor cells and 60% of cancer-associated fibroblasts (CAFs) cells, and accurately label mature red blood cells in the tumor microenvironment. In orthotopic transplanted pancreatic cancer models, the fluorescence intensity of GFNPs in tumors showed a positive correlation with the cycle size of tumor development. The differential spatial distribution of GFNPs in three typical clinical pancreatic cancer samples demonstrated their diagnostic potential. The complex of GFNPs and gemcitabine (GFNPS-GEM) nanoparticles has the advantages of in vivo stability, effective reduction of tumor size, and few side effects, which shows the potential for integrated diagnosis and treatment [84]. Wang et al. synthesized T1-T2 dual-mode imaging gadolinium doped iron oxide (GdIO) nanoclusters using iron acetylacetone and gadolinium acetylacetone as raw materials. In addition, in order to make the nanoclusters have targeted and antitumor effects, cyclic arginine glycine (cRGD) peptide and docetaxel (DTX) were attached to the surface of the nanoclusters, and the anti-pancreatic cancer effect of the modified nanoclusters was evaluated. The final synthesized material, cRGD-GdIO-DTX, actively targeted αvβ3 on the surface of Panc-1 pancreatic cancer cells. Compared with conventional passive targeting, the enrichment of cRGD-GdIO-DTX in tumor tissues improved, and the diagnostic accuracy was significantly enhanced. Moreover, the acidic tumor microenvironment triggered the release of DTX from cRGD-GdIO-DTX, thus achieving tumor treatment. The inhibition of the proliferation of SW1990 and Panc-1 pancreatic cancer cells by cRGD-GdIO-DTX was much stronger than that by the untargeted GdIO-DTX and free DTX in vitro. In a human pancreatic cancer xenograft model, cRGD-GdIO-DTX considerably slowed tumor development and demonstrated excellent magnetic resonance enhancement. The results suggest that cRGD-GdIO-DTX has potential applications for the precise diagnosis and efficient treatment of pancreatic cancer [85].

Although nanomaterials show promising prospects in experimental studies, they still face many challenges in clinical applications. First, the long-term safety of nanomaterials remains unclear, especially regarding the potential toxicity of their metabolic products in the body. The small size and unique morphology of nanomaterials result in behaviors within the body that are markedly different from those of traditional materials, which may lead to unexpected biological interactions. For instance, certain nanomaterials may accumulate in the body, potentially triggering immune responses or other toxic reactions. Therefore, assessing the long-term safety of nanomaterials requires systematic studies to ensure their safety in clinical applications.

Secondly, the large-scale production and quality control of nanomaterials poses difficulties. Compared to traditional materials, the synthesis of nanomaterials is often more complex, involving various chemical and physical methods. This not only increases production costs but may also lead to quality discrepancies between batches, affecting the reliability of clinical applications. To achieve the clinical translation of nanomaterials, standardized production processes and strict quality control systems must be established to ensure the consistency and safety of each batch of nanomaterials.

Furthermore, due to the inherent complexity and heterogeneity of pancreatic cancer, the clinical efficacy of nanomaterials may vary among individuals [86]. The biological characteristics of pancreatic cancer and individual patient differences can influence the distribution, metabolism, and therapeutic effects of nanomaterials. Therefore, in clinical applications, there are certain limitations in designing personalized treatment plans tailored to the specific circumstances of different patients.

Future research needs to further optimize the design of nanomaterials to enhance their targeting and biocompatibility. Moreover, verifying their safety and efficacy through clinical trials will be key to advancing breakthroughs in the clinical application of nanomaterials.

### 3.2. The Application of Nanomaterials in Pancreatitis Treatment

Pancreatitis is a severe inflammatory disease characterized by high mortality and recurrence rates caused by the self-digestion of the pancreas [5]. Nanomaterials, through enhancements such as responsiveness to reactive oxygen species and structural optimization, effectively deliver drugs but also exhibit anti-inflammatory, anti-fibrotic, and tissue repair-promoting functions, offering new hope for treating pancreatitis (as summarized in Table 3).

#### 3.2.1. Nanomaterials Aid Drug Delivery

Nanomaterials have been developed as carriers to load drugs for pancreatitis treatment. Apigenin, a compound with anti-fibrotic properties targeting pancreatic stellate cells (PSCs), has been successfully encapsulated into PEGylated PLGA nanoparticles for the treatment of chronic pancreatitis (CP). These nanoparticles measure approximately 160 nm in diameter and exhibit a high loading efficiency of 96 μg of apigenin per mg of nanoparticles. Research demonstrates that these apigenin-loaded nanoparticles effectively suppress PSC proliferation and induce apoptosis. To elucidate the therapeutic mechanism of apigenin, PSCs exhibiting pro-inflammatory and pro-fibrotic behavior were treated with the nanoparticles and analyzed using RT-PCR. The findings revealed a significant reduction in mRNA levels associated with inflammation and fibrosis, such as collagen 1A1, fibronectin, IL-6, and IL-8. In vivo studies further highlight the advantages of this delivery system, with PEGylated apigenin-loaded nanoparticles displaying prolonged circulation times in mice compared to free apigenin. Consistent with in vitro results, the in vivo experiments confirmed the nanoparticles’ anti-inflammatory and anti-fibrotic effects, demonstrated by the suppression of inflammation- and fibrosis-associated mRNA expression. The findings underscore the potential of nanomaterials to enhance drug delivery and efficacy [87]. Biomimetic nanoparticles prepared by cell membrane coating also exhibit excellent drug delivery performance. Chen et al. used macrophage membranes to encapsulate a PEG-PLGA and ulinastatin mixture, and a macrophage biomimetic nanoparticle was developed. This nanoparticle-based drug delivery system addresses issues related to low drug specificity and the difficulty of penetrating the blood–pancreas barrier. MU demonstrated excellent stability and biocompatibility in both in vitro and in vivo experiments. Additionally, MU exhibited strong inflammation-targeting capabilities in a subcutaneous inflammation model and an in situ pancreatitis mouse model. The results indicate that MU has a superior therapeutic effect on acute pancreatitis (AP) by inhibiting pro-inflammatory factors and maintaining cell viability [88]. Targeting the pathogenic trigger of phospholipase A2 (PLA2), Zhang et al. designed a delicate macrophage membrane-coated nanoparticle with polymeric cores wrapped with a natural macrophage membrane doped with melittin (PLA2 attractant) and MJ-33 (PLA2 inhibitor). This nanoparticle can synergistically lure and kill PLA2 enzymes, conferring effective protection against disease-associated inflammation, tissue damage, and lethality [89]. Nanomaterials play a crucial role as drug carriers in pancreatitis treatment. Through diverse structural designs, nanomaterials enhance drug delivery efficiency, improve bioavailability, and contribute to reducing inflammation and fibrosis.

#### 3.2.2. Nanomaterials as Anti-Inflammation Agents

The pathogenesis of acute pancreatitis is complex, involving the activation of pancreatic enzymes and the self-digestion of pancreatic tissue, leading to severe inflammatory responses. Nanomaterials, such as gold, selenium nanoparticles, and carbon nanoparticles, can effectively scavenge excess reactive oxygen species (ROS) due to their strong antioxidant capacity, thereby reducing inflammation. For example, gold nanoparticles, with their excellent antioxidant properties, can capture free radicals and reduce oxidative stress in cells, alleviating tissue damage caused by acute pancreatitis [90]. Selenium nanoparticles can inhibit the expression of inflammatory cytokines by modulating the NF-κB signaling pathway, thus reducing inflammatory responses [91]. Xie et al. reported that MoSe_2_-polyvinylpyrrolidone (PVP) nanoparticles (NPs) mimicked various natural enzymes (including catalase, peroxidase, superoxide dismutase, and glutathione peroxidase) and scavenged free radicals, with anti-inflammation effects on pancreatitis [92]. Fullerene, a potent antioxidant, can effectively scavenge ROS and free radicals, mitigating oxidative stress-induced cellular damage [93]. Moreover, macrophages play a central role in pancreatitis, and strategies that shift the M1 type (pro-inflammation) to M2 macrophages (anti-inflammation) are desirable for pancreatitis treatment. A study prepared carbon monoxide-bound hemoglobin vesicles (CO-HbVs) and found that they polarized macrophages to the M2 phenotype and suppressed neutrophil infiltration in the pancreas, alleviating pancreatitis [94]. Jing et al. constructed Ce and Gd bimetallic ion-doped CDs and prepared chitosan nanoparticles loaded with Ce/Gd-CDs and resveratrol (RES) and found that these nanoparticles could scavenge ROS and regulate macrophage polarization and imaging, significantly reducing acute pancreatitis [95]. These nanomaterials have shown great potential in the early treatment of acute pancreatitis and are expected to become a new direction for future pancreatic anti-inflammatory therapy.

#### 3.2.3. Nanomaterials Alleviate Pancreatic Fibrosis

Pancreatitis, particularly chronic pancreatitis, often leads to pancreatic fibrosis, which results in the gradual loss of pancreatic function and increases the risk of pancreatic cancer [6]. Pancreatic fibrosis is mainly manifested by an imbalance in extracellular matrix (ECM) homeostasis due to excessive deposition of collagen in the pancreas by activated pancreatic stellate cells (PSCs) [96]. Therefore, activated pancreatic stellate cells (PSCs) are the main source of collagen layer deposition and the key target in pancreatic fibrosis. The nano-system AT-CC offers an advanced approach to combating pancreatic fibrosis by precisely targeting activated pancreatic stellate cells (PSCs). Utilizing a bi-functional strategy, AT-CC integrates an anti-fibrotic drug with a collagen-targeting mechanism, enabling enhanced drug delivery to fibrotic tissues. The system’s unique design includes self-assembling lipid carriers that ensure stability and efficient drug release under pathological conditions. This targeted approach effectively inhibits the activation of PSCs and disrupts collagen deposition, the hallmark of fibrosis. Additionally, AT-CC significantly mitigates the progression of fibrosis through the downregulation of pro-fibrotic signaling pathways, including TGF-β and PDGFRβ axes. With its high therapeutic efficacy and minimal off-target toxicity, AT-CC demonstrates exceptional potential for clinical application in the treatment of chronic pancreatitis and related fibrotic disorders [97]. The LR-SSVA nano-system introduces an innovative therapeutic strategy for pancreatic fibrosis by combining dual-targeting and self-assembling capabilities to address the limitations of traditional drug delivery. This system leverages ligand-receptor interactions and a pH-responsive mechanism to ensure precise targeting of activated pancreatic stellate cells (PSCs) and effective drug release in fibrotic environments. The incorporation of self-assembling vesicles enhances stability during circulation while ensuring selective accumulation in the fibrotic pancreas. LR-SSVA achieves significant reductions in fibrosis by delivering therapeutic agents to PSCs, effectively inhibiting key pathways involved in fibrosis progression. Additionally, its multifunctional design enables deep tissue penetration, overcoming collagen barriers commonly found in fibrotic tissues. With superior biocompatibility and minimal off-target effects, LR-SSVA offers a groundbreaking approach to addressing pancreatic fibrosis, providing a potential foundation for treating other fibrotic disorders [59]. The nano-system LA-PC represents a promising advancement in the treatment of pancreatic fibrosis by specifically targeting pancreatic stellate cells (PSCs), which are pivotal in the development of this condition. Utilizing a “nanodrill” strategy, LA-PC enhances the delivery and accumulation of all-trans retinoic acid (ATRA) within PSCs. The system employs a PDGFRβ-targeting peptide (pPB) and collagenase to break down the collagen barrier that typically impedes drug delivery. This dual-action approach not only facilitates the penetration of the nano-system into fibrotic tissues but also significantly improves the drug delivery efficiency, achieving more than fivefold higher accumulation compared to free ATRA. Furthermore, LA-PC effectively inhibits the PDGF-BB/PDGFRβ axis by down-regulating the ERK pathway, thereby reducing fibrosis. This innovative platform offers a novel therapeutic strategy for precise regulation of PSC activity, highlighting its potential as a robust treatment option for chronic pancreatitis and possibly other fibrotic diseases [98]. Overall, nanomaterials offer innovative and targeted solutions to alleviate pancreatic fibrosis, addressing key challenges in drug delivery and fibrosis progression with enhanced precision and efficacy.

### 3.3. The Application of Nanomaterials in Diabetes Treatment

Diabetes is a chronic metabolic disorder resulting from insufficient insulin secretion or impaired insulin action, which leads to hyperglycemia and its related complications. Modified or drug-loaded nanomaterials facilitate the effective delivery of anti-diabetic drugs and possess the capability to improve insulin sensitivity, regulate blood glucose levels, and promote the regeneration of pancreatic β-cells, thereby offering new hope for diabetes treatment. Moreover, the targeting capabilities and biocompatibility of nanomaterials present promising opportunities for the prevention and treatment of complications associated with diabetes (as summarized in Table 4).

#### 3.3.1. Nanomaterials and Insulin Delivery

Diabetes is characterized by hyperglycemia, which is caused by the lack of insulin or insulin resistance due to the destruction of pancreatic islet cells [99]. Insulin, therefore, is a crucial hormone for regulating blood glucose levels. However, because of its protein nature, insulin is easily degraded by gastrointestinal enzymes, making traditional oral insulin ineffective. Traditional insulin therapy primarily relies on subcutaneous injections, necessitating frequent administration and strict blood glucose monitoring, which imposes significant inconvenience on patients [100]. Nanocarriers, innovative drug delivery systems, can deliver insulin via various routes, including oral, nasal, and transdermal methods. By encapsulating insulin, nanomaterials enable oral or sustained release, reducing injection frequency, enhancing patient compliance, and significantly improving insulin bioavailability [101]. Chitosan nanoparticles, with their excellent biocompatibility and biodegradability, can efficiently cross the gastrointestinal mucosal barrier, enhancing the absorption efficiency of oral insulin [102]. Their biocompatibility and sustained-release properties make them ideal carriers. Lipid and polymer nanoparticles are also widely utilized in oral insulin delivery, offering controlled drug release and ensuring more stable blood glucose levels [103]. A study prepared a crystalline mesoporous metal–organic framework (MOF) NU-1000 and immobilized insulin in it with a high loading of ~40 wt%. This MOF material was stable in a simulated stomach environment, being a potential insulin carrier for oral delivery [104]. Another study reported a microalgae-based oral insulin delivery strategy (CV@INS@ALG) using Chlorella vulgaris (CV) cross-linked with sodium alginate (ALG). This hydrogel could overcome the gastrointestinal barrier, protect insulin from harsh gastric conditions, and achieve a pH-responsive drug release in the intestine [44]. Nanotechnology is also being integrated into closed-loop insulin delivery systems, which, in conjunction with blood glucose monitoring devices, adjust insulin release in real-time for more precise blood glucose control [105]. Additionally, in nasal delivery, nanomaterials, due to their ultra-small size and excellent adhesion, can effectively traverse the nasal epithelial barrier, enabling rapid insulin absorption and bypassing the hepatic first-pass effect. This non-invasive delivery method facilitates relatively rapid blood glucose regulation, making it suitable for managing acute hyperglycemia. Finally, the application of nanomaterials in transdermal insulin delivery has garnered significant attention. It can deliver drugs directly through the skin, avoiding gastrointestinal degradation and hepatic first-pass effects, thereby improving the bioavailability of drugs. It is suitable for various applications such as vaccination, drug delivery, monitoring, and diagnosis. Encapsulating insulin within nanocarriers, coupled with microneedle technology, enables painless and sustained insulin release [106]. This method not only enhances patient compliance but also mitigates skin discomfort and infection risks associated with frequent insulin injections.

#### 3.3.2. Nanomaterials for the Protection of Islet Cells

The onset and progression of diabetes are closely linked to islet cell dysfunction, particularly in type 1 diabetes, where the autoimmune destruction of pancreatic β cells results in insufficient insulin secretion. Nanomaterials, due to their unique physicochemical properties, can safeguard islet cells through multiple mechanisms. Antioxidant nanomaterials, such as fullerenes, can alleviate oxidative stress damage to pancreatic cells by scavenging excess reactive oxygen species (ROS), thereby alleviating pancreatic compensatory hyperplasia [107]. Selenium nanoparticles, with their exceptional antioxidant properties, help mitigate oxidative stress in islet cells by scavenging free radicals [108]. Other metallic nanoparticles (gold, silver, and copper) and metallic oxide (ZnO) have been reported to exhibit anti-diabetic effects, possibly via reducing oxidative stress and regulating antioxidant defense, regulation of glucose utilization, and insulin sensitivity [109]. In addition, nanoparticles have been shown to be perfect carriers for the delivery of anti-diabetes drugs. Wang et al. prepared a glucagon-like peptide 1 (GLP-1)-modified polyethyleneglycol-polycaprolactone (PEG-PCL) nanoparticle to load mangiferin (MGF), finding they could increase β-cell proliferation, reduce β-cell apoptosis, and repair islet cells [57]. As an autoimmune disease, type 1 diabetes can be treated with the immunomodulatory strategy, which can reduce immune-mediated damage to the pancreatic islet cells. Nanoparticles can deliver general immunosuppression, biological drugs, tolerogenic agents, and autoantigens. Jung et al. developed MECA79 mAb-modified poly(lactic-co-glycolic acid)–poly(ethylene glycol) nanoparticles encapsulated with anti-CD3 mAb, which could target both the PLNs and pancreata of NOD mice and release free anti-CD3 to induce T cell anergy and/or apoptosis, thereby shifting the immunologic balance toward immunoregulation [58]. Basarkar et al. prepared poly(lactide-coglycolide) (PLGA) and methacrylate copolymer nanoparticles to deliver plasmid DNA-encoding mouse interleukin-10, preventing autoimmune diabetes [110]. Au et al. described a strategy using pretargeting and glycochemistry to bioengineer β cells in situ to enhance β-cell-specific tolerance. They first delivered the β-cell-targeted Ac4ManNAz-encapsulated nanoparticles to the surface of pancreatic β cells and then injected dibenzylcyclooctyne (DBCO)-functionalized programmed death-ligand 1 immunoglobulin fusion protein (PD-L1-Ig) to conjugate to the surface of native β cells, inducing antigen-specific tolerance. This strategy utilizes bioorthogonal stain-promoted azide–alkyne cycloaddition to selectively conjugate PD-L1 onto β cells in vivo. By simultaneously presenting islet-specific antigens and PD-L1 to engaged T cells, this approach reverses early-onset type 1 diabetes mellitus (T1DM) by reducing IFN-gamma-expressing cytotoxic T cells and promoting antigen-specific immune tolerance [111]. A study developed nanoparticles coated with autoimmune-disease-relevant peptides bound to major histocompatibility complex class II (pMHCII) molecules, which triggered the generation and expansion of antigen-specific regulatory CD4+ T cell type 1 (TR1)-like cells. These TR1-like cells suppressed autoantigen-loaded antigen-presenting cells and drove the differentiation of cognate B cells into disease-suppressing regulatory B cells. Importantly, this approach resolved established autoimmune phenomena in various mouse models, including mice humanized with lymphocytes from patients, without compromising systemic immunity. These findings highlight pMHCII-based nanomedicines as a promising new class of drugs for treating a broad spectrum of autoimmune diseases in a disease-specific and genetically independent manner [112].

#### 3.3.3. The Role of Nanomaterials in Improving Insulin Resistance

Insulin resistance, a key characteristic of type 2 diabetes, is marked by the reduced sensitivity of body cells to insulin, leading to ineffective blood glucose regulation. Nanomaterials can enhance insulin sensitivity through various mechanisms. For instance, functionalized fullerenes have been shown to decrease the release of inflammatory factors, directly exerting anti-inflammatory effects that contribute to enhancing insulin sensitivity. Similarly, gadofullerene nanoparticles enhance insulin sensitivity by modulating oxidative stress and inflammatory responses, thereby assisting in the control of blood glucose levels [63,113]. Silver and zinc oxide nanoparticles have demonstrated significant anti-inflammatory effects, reducing insulin resistance through the modulation of cytokine expression. These nanoparticles enhance the phosphorylation of insulin receptor substrate-1 (IRS-1), activate the phosphoinositide 3-kinase (PI3K)/protein kinase B (Akt) pathway, promote the translocation of glucose transporter-4 (GLUT4), and increase glucose uptake, thereby improving insulin resistance [114]. Additionally, except for abnormal changes in blood glucose, lipid metabolism disorders are also closely related to insulin resistance. Specific nanomaterials can enhance insulin signaling pathways by modulating lipid metabolism, thereby reducing insulin resistance and ultimately playing a role in the treatment of type 2 diabetes [115]. Hang et al. developed a drug delivery system utilizing self-assembling polymer-amino acid conjugates (γ-PGA-PAE) to achieve sustained release of the GLP-1 analog DLG3312. This nano delivery system not only provided effective encapsulation of DLG3312 but also had a relatively stable structure. This approach ensures a controlled release profile, which is crucial for maintaining therapeutic levels of DLG3312 over an extended period [116]. This approach combined the molecular and materials engineering strategies that offered a unique solution. These applications of nanotechnology in diabetes treatment not only improve drug efficacy but also reduce the side effects of traditional treatments.

## 4. The Prospects for Nanomaterial-Based Treatment of Pancreatic Diseases

With advancing technology, emerging trends in the application of nanomaterials for pancreatic disease treatment continue to evolve. These trends drive both the advancement of basic research and the creation of new opportunities in clinical applications. From early diagnosis and personalized treatment to real-time monitoring, nanotechnology has significantly advanced research and clinical practice for pancreatic diseases. The continuous emergence of multifunctional nanomaterials, the integration of gene editing with nanotechnology, the development of immuno-nanomaterials, and the integration of microbiome and nanotechnology will further deepen the understanding of nanomaterials in pancreatic diseases and enhance their clinical translation [117]. These efforts are anticipated to significantly enhance patient prognosis in pancreatic diseases and elevate the field of nanomedicine.

### 4.1. Nanomaterials Combine with Omics to Aid Precision Medicine

With the advancement of precision medicine, personalized treatment has increasingly become a forefront topic in medical research. Due to the complex pathogenesis and heterogeneity of pancreatic diseases, traditional one-size-fits-all treatment approaches often result in suboptimal outcomes [118]. Nanotechnology, with its highly customizable properties, provides novel approaches for the personalized treatment of pancreatic diseases. In personalized treatment, gene testing and proteomics analysis are two key steps [119]. Gene testing can identify mutated genes or abnormal gene expressions associated with pancreatic diseases in patients. Proteomics analysis aids in understanding the protein expression levels in patients and their correlation with their disease. By integrating this information with nanotechnology, researchers can develop personalized treatment plans tailored to individual patients. For example, depending on the patient’s gene mutation type, specific nanocarriers and drugs can be selected to precisely deliver the drug to the diseased tissue, enhancing treatment efficacy while minimizing side effects [120]. In the future, as genomics, proteomics, and nanotechnology continue to advance, personalized treatment plans will become more refined. Through integrating various “omics” data with advanced nanotechnology, healthcare professionals can tailor treatment plans that best suit each patient’s condition, thereby achieving true precision medicine.

### 4.2. Nanomaterials Incorporate Emerging Technologies Assist Pancreatic Diseases Treatment

Multifunctional nanomaterials can integrate diagnosis, treatment, and monitoring functions into a single nanoplatform, enabling “one-stop” disease management [99,121]. A typical application of multifunctional nanomaterials involves the development of a “theranostic” nanoplatform. This platform can encapsulate both imaging probes and therapeutic drugs within nanoparticles, allowing for simultaneous disease diagnosis and treatment [122]. This “theranostic” platform is highly significant for the early detection and precise treatment of pancreatic diseases, significantly improving patient survival rates. Therefore, the integration of stimulus-responsive functional elements through rationally designed nanocarriers is valuable for precise triggering by specific biomarkers or external stimuli (such as light, temperature, or pH levels) [48,123]. Due to the heterogeneity of diseases and individual differences, there are still many issues that need to be addressed to promote the clinical translation of “theranostics” nanomaterials. In addition, ensuring that nanomaterials balance safety and efficacy in vivo is important for the actual therapeutic process. Meanwhile, the preparation process of nanomaterials is complex, and addressing the feasibility and consistency of large-scale production remains an urgent issue.

Gene editing technologies, like the CRISPR/Cas9 system, offer powerful tools for precision treatment. Encapsulating the CRISPR/Cas9 system within nanocarriers can effectively protect the stability of gene editing tools in vivo and improve their delivery efficiency to target cells. In pancreatic cancer treatment, researchers successfully used lipid nanoparticles to deliver the CRISPR/Cas system to pancreatic tumor cells, resulting in the knockout of oncogenes, including *KRAS* and *TP53*, and significant inhibition of tumor growth [124]. The fusion of gene editing systems with nanotechnology provides new avenues for the precision treatment of pancreatic diseases. However, gene editing systems suffer from off-target effects, so it remains challenging to construct safe and effective nanocarriers to avoid toxic side effects caused by off-target effects. That is, the constructed nanocarriers are not only stable and protective of their loaded gene editing systems but also can facilitate their efficient delivery to target cells. Currently, the main focus of constructed clinically translational nanomaterials is still on avoiding the removal of physiological barriers in vivo, improving target site delivery efficiency, and maximizing therapeutic efficacy at low doses.

Immunotherapy has made breakthrough progress in cancer treatment in recent years. Nanotechnology presents new opportunities for enhancing immunotherapy outcomes, which can deliver immune checkpoint inhibitors like PD-1 or CTLA-4 antibodies to pancreatic tumor cells, enhancing the killing effect of T cells [125]. Although nanomaterials can enhance the delivery efficiency of immune checkpoint inhibitors, the complex microenvironment of pancreatic cancer contributes to the ability of tumor cells to evade the attack of the immune system through multiple mechanisms. Therefore, optimizing nanocarriers to modulate both the tumor microenvironment and deep tissue penetration remains a top priority for pancreatic cancer therapy. Activation of the patient’s own immune system, in particular, is expected to prolong their survival through various means.

The role of the microbiome in cancer and metabolic diseases has garnered significant attention. Studies have shown that the gut microbiome is closely related to the occurrence and progression of pancreatic diseases. Nanotechnology can modulate the gut microbiome, thereby influencing the progression of pancreatic diseases. Nanoparticles can deliver probiotics or prebiotics to the gut, regulating the gut microbiota composition and influencing the pathological processes of pancreatic diseases [126]. This emerging field offers novel perspectives and strategies for treating pancreatic diseases. However, effectively modulating the microbiome while ensuring its safety and efficacy in clinical applications requires substantial experimental and clinical data support. Overall, although modulating intestinal flora to treat pancreatic diseases has a promising future, how to articulate a clear mechanism of action between the intestinal–pancreatic axis, as well as the beneficial effects of certain flora or prebiotics on the pancreas specifically, still needs to be explored by more researchers.

## 5. Conclusions

In summary, this review highlights the transformative potential of nanomaterials in the treatment of pancreatic-related diseases, emphasizing their role as advanced tools for overcoming traditional therapeutic limitations. By leveraging their unique physicochemical properties, nanomaterials have demonstrated an exceptional capacity to enhance pancreatic targeting and improve therapeutic efficacy while minimizing side effects. Through structural optimization, surface modifications, and intelligent responsiveness, these materials address key challenges in drug delivery, ensuring higher therapeutic concentrations at target sites, improved bioavailability, and reduced systemic toxicity. Furthermore, the versatility of nanomaterials enables their application across a wide spectrum of pancreatic diseases. In pancreatic cancer, they serve as effective drug delivery platforms, enhance the efficacy of radiation therapy, and act as multifunctional theranostic tools, offering comprehensive solutions for complex treatment challenges. For pancreatitis, nanomaterials provide dual benefits as both drug carriers and therapeutic agents, playing a significant role in reducing inflammation, alleviating fibrosis, and promoting tissue repair. In diabetes management, these materials offer innovative solutions for insulin delivery, β-cell protection, and improvement of insulin resistance, as well as support for islet transplantation, marking significant strides in addressing this chronic condition.

Beyond their therapeutic roles, nanomaterials present broader implications by serving as multifunctional platforms that integrate treatment and diagnostics into unified systems. This capability ensures a holistic approach to disease management while also enhancing the efficiency and precision of current therapeutic modalities. Furthermore, their integration with emerging technologies such as omics and bioinformatics aligns with the evolving landscape of precision medicine, opening new avenues for tailored and personalized treatments. However, some technical and safety problems of nanomaterials remain to be solved before their widespread clinical application. Future research should focus on optimizing the design and functionality of nanomaterials, enhancing their biocompatibility and therapeutic efficacy to achieve safer and more effective treatment strategies, and the preparation controllability and stability of the nanomaterials should be determined. Additionally, larger-scale clinical trials are essential to validate their potential applications.

Looking ahead, the future of nanomaterial applications in pancreatic disease treatment appears promising, especially as they synergize with advanced technologies to deepen our understanding of disease mechanisms and enable the development of next-generation therapeutic strategies. Efforts to create sustainable and biocompatible nanomaterials will further address concerns related to toxicity and long-term effects, ensuring broader clinical applicability. Altogether, the insights presented in this review underscore the immense potential of nanomaterials in revolutionizing the management of pancreatic diseases, offering enhanced treatment efficacy, reduced systemic toxicity, and expanded therapeutic options. These advancements not only reflect current progress but also pave the way for innovative solutions that will shape the future of advanced medical interventions.

## Figures and Tables

**Table 1:** Nanomaterials for enhanced pancreatic targeting via surface modification and structural optimization.

Route	Nanomaterials	Target Mechanism	Advantages
Oral Administration	CB-MCs@INS	PAT1	Protect insulin from the invasion of gastric acid and overcome mucus and epithelial barriers.
	CV@INS@ALG	pH response and endocytosis	Overcome the gastrointestinal barrier and achieve a pH-responsive drug release in the intestine.
	ACU@BI	AMPK/AKT/IRS-1	Overcome multi-intestinal barriers and extend insulin treatment duration.
	PLGA-TCA/DLPC/HCQ	Apical sodium-dependent bile acid transporter-mediated and lymphatic transportation	Enhance oral absorption and pancreas accumulation properties.
Intravenous Injection	BP-GEM@NPs	iRGD	Induce much more pancreatic tumor cell apoptosis and synergistically inhibit tumor growth in both subcutaneous xenograft and orthotopic models.
	MGF/GDPP	GLP-1 receptor	More concentration in the pancreas, with better blood glucose and glucose tolerance, enhances insulin levels, increases β-cell proliferation, reduces β-cell apoptosis, and islet repair in vivo.
	MECA79-anti-CD3-NPs	HEV	Improve accumulation of anti-CD3 mAb in both the PLNs and pancreata of NOD mice.
	BAPTA-AM	ICAM-1 and PACs	Exhibite efficient recruitment at the inflammatory endothelium, trans-endothelial migration, and precise acinar cell targeting, resulting in rapid pancreatic localization and higher accumulation.
	LR-SSVA	PSC and ROS response	Reverse the imbalanced ECM homeostasis to ameliorate pancreatic fibrosis.
Intraperitoneal Injection	UCNP@PC	Cell membranes	A 16-fold improvement in the efficacy of utilization promotes intraperitoneally administered UCNP@PC in monitoring orthotopic pancreatic cancer compared with the IV approach.
	PAMD-CHOL/siRNA	CXCR4 and PLK1	Increase tumor accumulation and deeper penetration into the tumors.
	HGN	IPT-BT	Effectively cause double-stranded DNA damage and improve the oxygen supply and the penetration of nanoparticles inside the tumor.
	ATF-PEG-IONPs	uPAR	Efficiently penetrates into both the peripheral and central tumor areas in the primary tumor as well as peritoneal metastatic tumor.
	DOTAP-containing LNPs	Peritoneal macrophage exosome	Lead to robust and specific protein expression predominantly in insulin-producing β cells of the pancreas.

**Table 2:** Applications of nanomaterials in pancreatic cancer treatment.

Optimization of Treatment	Nanomaterials	Advantages	Limits
Improve drug delivery efficiency	TAB004-NPs	Increased accumulation in the PDA tumor compared to the non-conjugated nanocarrier.	Unvalidated in vivo models of therapeutic efficacy and the challenge of cancer cell resistance to chemotherapy drugs.
	FAD-Dox	Potent anticancer activity, overcoming the heterogeneity of drug response, no adverse reactions, with good tolerance.	Remaining in large-scale production feasibility and maintaining consistency and stability in the production process.
	KRAS-siRNA NP	High uptake rate of cancer cells, enabling the efficient delivery of KRAS-specific siRNA to suppress KRAS expression and significantly decrease cell viability.	The effect of in vivo experimental therapy is not stable, and there may be an off-target effect of siRNA targeting KRAS, and the delivery rate and specificity in clinical application still need to be further optimized.
	CMS/PEG-B-P	Reshape the tumor microenvironment and transform the immunosuppressive type into an immune-promoting type, thus enhancing immunotherapy.	Maximizing the efficacy of combining CXCR4 and PD-L1 inhibitors, further optimization of drug combinations and dosing is needed to ensure the best therapeutic outcome without increasing toxicity.
Enhance the efficacy of radiotherapy	GDNDs	Enhances the effect of radiotherapy by absorbing incident light and converting it into heat	Multifunctional GDNDs need to be further studied, and the cost of precious metals is high.
	CONPs	Sensitize pancreatic cancer cells to RT and protect normal tissues from harmful side effects.	The selectivity and mechanism of its action still need to be further studied.
	Col-TNP	Through enzymatic collagen-degrading capability and tumor-responsive surface charge reversibility, achieves an ECM-tampering effect.	The complexity of the production and preparation process limits the feasibility of large-scale production and quality control.
Integration of diagnosis and treatment	GFNPS-GEM	Effectively diagnose and combine clinical chemotherapy drugs in various ways.	Insufficient drug loading efficiency and controlled release.
	cRGD-GdIO-DTX	Considerably slowed tumor development and demonstrated excellent magnetic resonance enhancement.	Potential cytotoxicity and immune reactions may occur at prolonged and high doses of exposure.

**Table 3:** Applications of nanomaterials in pancreatitis treatment.

Optimization of Treatment	Nanomaterials	Advantages	Limits
Drug-loaded carriers	Apigenin-loaded PLGA nanoparticles	Reduces the expression of mRNAs associated with PSC pro-inflammation and pro-fibrosis and has a longer circulation time in mice.	Insufficient targeting and controlled release of drugs.
	MU	A great inflammation targeting effect and inhibited pro-inflammatory factors and keeping cells viability.	Controllability of drug release and drug loading efficiency.
	MΦ-NP(L&K)	Provides a bionic therapeutic strategy to inhibit PLA2-induced inflammatory response.	The effectiveness of targeting and the stability of complex nanoparticles.
Enhance anti-inflammatory	Selenium nanoparticles	Lessens the developed pancreatic injury, and it may be used in prevention of acute pancreatitis and the associated hyperglycemia in vulnerable individuals.	Dose control and maintain safe and effective concentrations, especially in different disease models and individuals.
	MoSe2-PVP NPs	Mimic natural enzymes and effectively scavenge free radicals, targeting mitochondrial and intracellular ROS and RNS to ameliorate AP.	Light penetration depth is limited in clinical applications of photothermal/photodynamic therapy.
	CO-HbVs	Regulation of macrophage and neutrophil activity.	Lack of accurate control of release and rate excess co can lead to severe toxicity.
Inhibit pancreatic fibrosis	LR-SSVA	Selective targeting normalizes ECM homeostasis through ROS response-controlled release of drugs.	The preparation process is complicated, and there are potential immune reactions.
	AT-CC	Strong targeting, dual drug loading specific therapy.	Stability and controlled drug release in the environment of complex organisms.
	LA-PC	High selective targeted drug delivery efficiency Dual internal and external regulatory strategies inhibit activation of PSCs.	The complex preparation process, long-term performance, and stability need to be further verified.

**Table 4:** Applications of nanomaterials in diabetes treatment.

Optimization of Treatment	Nanomaterials	Advantages	Limits
Improve insulin delivery efficiency	(MOF) NU-1000	Drug delivery stability and high load rate in a short time.	Precise delivery and controlled drug release.
	MNs	Painless drug delivery and improved insulin bioavailability.	Limited drug loading capacity and challenges in long-term storage stability and skin impact from prolonged use.
	CV@INS@ALG	Dual delivery mechanism improves insulin absorption efficiency and enhances insulin sensitivity through long-term oral administration.	Long-term storage stability and individual gastrointestinal tract differences.
Protect pancreatic cells	Metallic nanoparticles	Excellent antioxidant and anti-inflammatory effects and are a good drug carrier.	Toxicity control and biodegradability issues.
	MGF/GDPP	Specific recognition of pancreatic β cells and delivery of antioxidant and anti-inflammatory drugs.	Unclear metabolic and clearance pathways and long-term in vivo safety.
	MECA79-anti-CD3-NPs	Highly targeted, precise delivery reduces systemic side effects and can enhance immune regulation.	Long-term safety and biological distribution of metabolic pathways and production process complexity.
	PLGA/E100 nanoparticles	Improving insulin delivery stability enhances its bioavailability and achieves sustained release.	In vivo distribution, metabolism, and clearance pathways are uncertain, and the preparation process is complex.
	Ac_4_ManNAz NPs	Specifically targeting and contributing to immune regulation and modification of beta cells in vivo.	The immunogenic risks and unknown long-term effects and preparation process are complex.
	pMHC–NPs	Specifically targeted and involved in immunity from regulation and protection of beta cell function.	Delivery efficiency and long-term efficacy.
Enhance insulin sensitivity	Functionalized fullerenes	Target specific tissues or cells and have good antioxidant capacity and anti-inflammatory properties.	High production costs and unclear metabolic and clearance pathways.
	Silver and Zinc oxide nanoparticles	Antioxidant and anti-inflammatory properties, along with exhibiting significant hypoglycemic effects.	The mechanism of action has not been fully elucidated, and the understanding of metabolic clearance pathways is limited.
	DLG3312@NPs	High drug-loading efficiency and sustained drug release with significant blood glucose regulation effects.	High production costs and the lack of detailed molecular mechanisms for enhancing insulin sensitivity.
